# Perceptions and practices of Swedish wild boar hunters in relation to African swine fever before the first outbreak in Sweden

**DOI:** 10.1186/s12917-024-04183-9

**Published:** 2024-07-17

**Authors:** Erika Chenais, Linda Ernholm, Annie Frisk Brunzell, Karl Mård, Lotta Svensson, Johanna F. Lindahl, Susanna Sternberg Lewerin

**Affiliations:** 1 Swedish Veterinary Agency, Uppsala, 751 89 Sweden; 2 Swedish University of Agricultural Science, Uppsala, 750 07 Sweden; 3 Evidensia djurkliniken i Nacka, Nacka, Sverige; 4 Distriktsveterinärerna Borensberg, Borensberg, Sweden; 5 Billdals djurklinik Evidensia, Billdal, Sverige

**Keywords:** Focus group discussions, Questionnaire, Disease control, Hunting tourism

## Abstract

**Background:**

The first outbreak of African Swine Fever (ASF) in Sweden was detected in 2023 in wild boar. This study was conducted before the first ASF outbreak with the objective of investigating Swedish hunters’ perceptions and practices pertaining to ASF ahead of any potential future outbreak.

A mixed-methods interview study with Swedish wild boar hunters, consisting of focus group discussions and a questionnaire, was undertaken between October 2020 and December 2021. Six focus groups were conducted online, and an online questionnaire with questions related to practices and habits concerning hunting, the use of bait and hunting trips was sent to all members of the Swedish Hunting and Wildlife Association. A total of 3244 responses were received.

**Results:**

Three general themes were identified in a thematic analysis of the data from the focus groups: hunters are willing to engage in ASF prevention and control, simplicity and feasibility are crucial for the implementation of reporting, sampling and control measures, and more information and the greater involvement of the authorities are required in ASF prevention and control. Results from the questionnaire showed that the use of bait was common. Products of animal origin were rarely used for baiting; the most common product used was maize. Hunting trips abroad, especially outside of the Nordic countries, were uncommon.

**Conclusions:**

Hunting tourism and the use of bait do not seem to constitute a major risk for the introduction of ASF to wild boar populations in Sweden. The accessibility of relevant information for each concerned stakeholder and the ease of reporting and sampling are crucial to maintain the positive engagement of hunters.

**Supplementary Information:**

The online version contains supplementary material available 10.1186/s12917-024-04183-9.

## Background

The incursion of African swine fever (ASF) into Georgia in 2007 [1] was the starting point of the current epidemic of ASF in Europe. Since then, the epidemic has developed in unprecedented global dimensions [2]. The disease is currently present in large parts of Europe (to date: Azerbaijan, Bosnia and Herzegovina, Belarus, Belgium (declared free in 2020), Bulgaria, Croatia, Czech Republic, Estonia, Georgia, Germany, Greece, Hungary, Italy, Latvia, Lithuania, Moldova, North Macedonia, Poland, Romania, Russia, Serbia, Slovakia, Sweden and Ukraine), and the continuous spread, emergence and re-emergence of ASF present a constant threat to domestic pigs and wild boar [3, 4]. Controlling ASF in wild boar populations has proved difficult [5]. In the current epidemic in Europe, only Belgium and the Czech Republic have so far managed to eradicate the disease after its introduction into a wild boar population (the Czech Republic was re-infected in 2022) [6, 7]. Hunters have been identified as extremely important stakeholders in ASF control [8, 9] and several studies have investigated European hunters’ perspectives in relation to the disease [10–12]. However, as hunting realities and practices, land ownership and wild boar population dynamics vary between countries, it is important to understand their perspectives in a local context [13]. For the same reason, data related to the risks of introducing ASF into new areas and the further spread of the disease in these areas need to be collected locally [14].

Hunting tourism and certain hunting practices have been identified as risk factors for introducing or spreading ASF [15]. To hunt in a foreign country the individual hunter needs follow the country's rules for weapons and for hunting, and if bringing a hunting weapon, have a weapon’s license and a permit for travelling with the weapon (weapon passport). To hunt in Sweden a valid hunting licence and hunting card (issued from the Swedish Environmental Protection Agency) is needed. Access to hunting grounds must be given by the landowner. Most Swedish hunters belong to local hunting groups and are members of either of the two hunters’ organisation, the Swedish Association for Hunting and Wildlife Management (SJF) being the largest with > 150,000 members or the Hunters’ National Association with about 40,000 members.

The first outbreak of ASF in Sweden occurred in September 2023 in wild boar in an area at the northern limit of the bioregion for wild boar [16], where there is a relatively low wild boar density and few domestic pig holdings [17]. The exact source of the outbreak has not been identified, but disease introduction through natural wild boar movements was ruled out as most areas containing wild boar populations in Sweden are surrounded by water, preventing direct contact between Swedish wild boar and ASFV-infected populations in neighbouring countries. The only area that has a wild boar population and a land border is the western part of Sweden, which borders Norway. Norway has a very limited wild boar population, which is free of ASF. It was assumed that the virus reached the wild boar population via virus-contaminated food waste from domestic pigs or wild boar in an affected country [17]. Swedish pig farmers’ perceptions of this outbreak have been described [18], and the perceptions and experiences of the hunters who participated in outbreak control actions are currently being investigated. However, general information about Swedish hunters’ knowledge, attitudes and practices in relation to ASF prior to the outbreak, has not yet been compiled. This study was conducted before the first ASF outbreak in Sweden with the objective of investigating the perceptions and practices of Swedish hunters ahead of any potential future outbreak in order to be able to make use of the lessons learned should ASF come to Sweden.

## Methods

This interview study with Swedish wild boar hunters was implemented between October 2020 and December 2021 and consisted of focus group discussions (FGD) and a questionnaire. The methods for these two parts are described separately below.

### Focus group discussions

FGDs were conducted online using video conference software (Zoom Video Communications, Inc., San Jose, California, United States) in October and November 2020.

#### Study area and participant selection

**Fig. 1:**
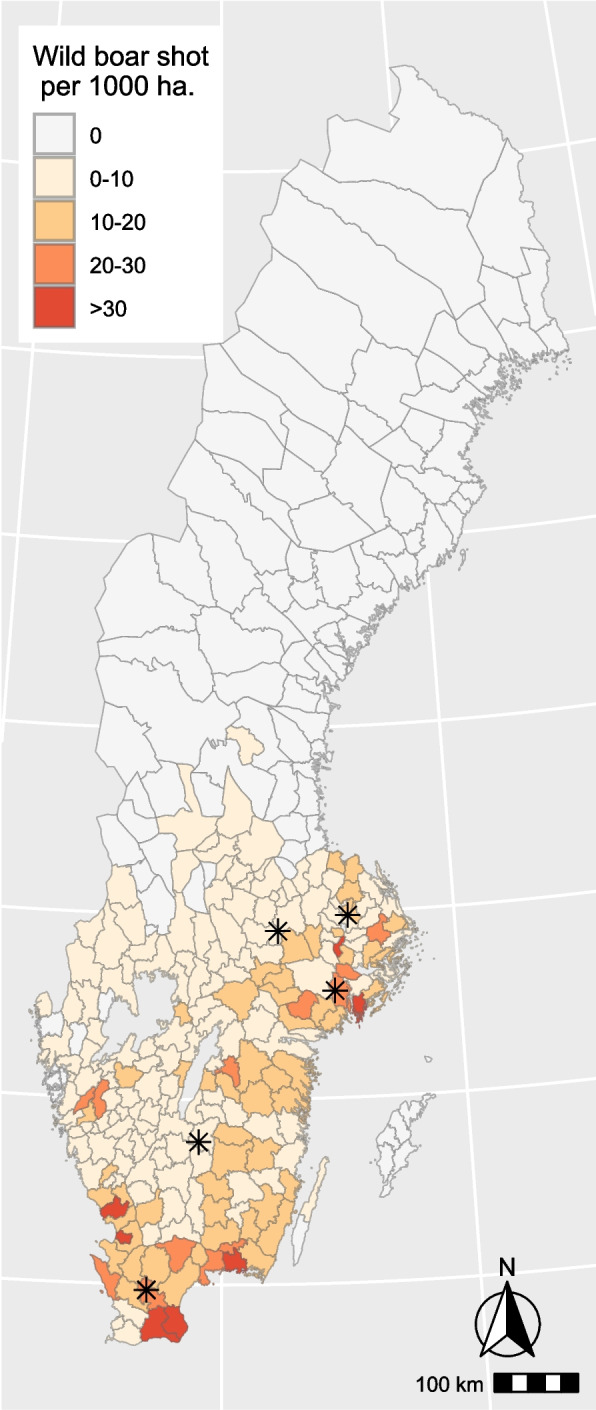
Map showing numbers of wild boar shot per 1000 ha in Sweden 2018–2019. The distribution of the hunting bag is considered to reflect the distribution of the wild boar population. Asterisks represent the approximate locations of the hunting districts of participants in the focus group discussions. The map was created in the statistical software ‘R’ (R core team, Vienna 2024), using the package ‘ggspatial’. Data source: “The Swedish Association for Hunting and Wildlife Management, game monitoring. Available online: www.viltdata.se”

Based on an evaluation of the geographical distribution of the wild boar population in Sweden, the decision was taken to limit the study area for the FGDs to southern/central Sweden (Fig. 1). Interviewees were recruited with the help of local representatives of SJF in the study area, with the inclusion criteria being people who hunted wild boar in Sweden and were aged 18 and over. Membership in SJF was not an inclusion criterion and not asked for or recorded. Discussions were arranged in hunting districts where at least three hunters would be willing to participate in an online FGD. To facilitate an inclusive and participative discussion, and especially as the FGDs took place online, it was decided to include maximum five participants per group. Once at least three people had agreed to participate, an invitation was sent by email. Additional groups were included until data saturation was achieved, meaning that no new information emerged from the discussions.

#### Data collection

The FGDs were conducted in Swedish and led by a facilitator (AFB or LS), and followed a topic guide (see Additional file 1). Before the first FGD, the topic guide was tested in a pilot FGD and adapted accordingly. Each FGD started with the facilitator introducing the study and the research team and informing participants about data handling and confidentiality. With the consent of all participants, the discussions were recorded via the built-in recording feature in the video conference tool, with detailed notes taken as backup. Recordings were transcribed *ad verbatim.* Following the introduction, a short presentation was given by one of the researchers (EC or LE) about the current situation regarding ASF in Europe and in Sweden, before the group discussion took place. The FGDs were flexible, allowing the discussion to evolve according to the participants’ interests and priorities, while the facilitators ensured that the topics in the topic guide were covered. When the discussion concluded, the second part of the presentation was given, focusing on the prevention and control of ASF. At this point in the meetings, participants could ask any questions arising out of the discussion. Each FGD lasted approximately two hours.

#### Data analysis

Transcripts were imported into a qualitative data analysis software (NVivo, QSR International Pty Ltd. Version 12, 2018) and coded. At all steps of the analysis, codes and themes were allowed to emerge inductively through repeated reading of the data, with the aim of capturing the participants’ perspectives. Based on primary codes representing similar expressions and reasoning, emerging themes and general overarching topics were developed. The analysis was performed in Swedish and, once established, the codes, themes and topics were translated into English. Where participants are quoted, their answers have been translated into English.

### Online questionnaire

The online questionnaire was created in the software survey tool Netigate (Netigate AB, Stockholm, Sweden) and was available to respondents during the month of November 2021.

#### Data collection

The online questionnaire was written in Swedish and had 28 single response or multiple-choice, closed or semi-closed questions focusing on wild boar hunting, and related to hunting practices, hunting travels and the use of bait. An English version of the final questionnaire, translated for the purposes of this article, is included in Additional file 2. The replies were anonymous and no personal information, such as age, gender or home location, was collected.

A pilot version of the questionnaire was tested on a group of people that were active hunters or that had former hunting experience and adapted accordingly. Subsequently, a first version was distributed through the authors’ personal contacts, hunting groups on social media, and via the website of the Swedish Veterinary Agency (SVA). Based on feedback from the respondents of the first version, a second, slightly adapted and improved version was distributed to all members of the SJF who had a registered e-mail address. There was no selection based on region or whether the receiver had an active hunting permit. This second version of the questionnaire was distributed in early November 2021 and remained open during that month. To avoid duplicate answers, only responses from the second version were included in this study.

#### Data analysis

**Fig. 2:**
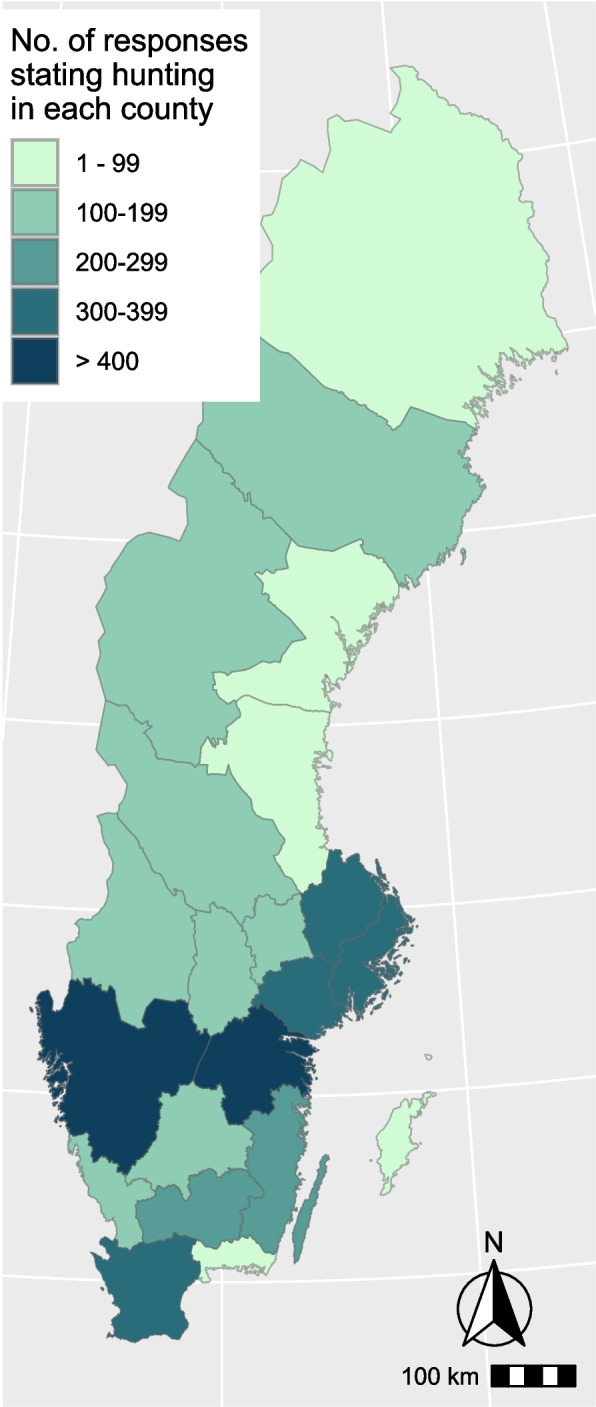
Map visualising the county/counties in which the respondents hunt. As this was a multiple-choice question, respondents may have selected more than one county as the numbers exceed the total number of questionnaire responses

Questionnaire data were exported from the Netigate tool in Excel format. Further handling and analysis were performed in the open-source statistical program R (R Core team, 2022). Graphs were made with ‘ggplot’ from the ‘tidyverse’ package, and the map in Fig. 2 was created using the ‘ggspatial’ package.

The words ‘baiting’ and ‘supportive feeding’ were used either in conjunction or interchangeably throughout the questionnaire. For the purpose of this analysis, the two concepts were considered as the same practice of ‘baiting’, i.e. placement of feed in order to attract wild animals. When relevant, categorical answers were aggregated to accommodate the analyses. Free-text answers, given if the respondent was asked to specify a selected answer further, were read and analysed for content and used to improve and deepen understanding of the quantitative data.

Whenever appropriate, descriptive statistics were produced and associations between categorical variables were assessed by Pearson’s chi-squared test or odds ratio calculations. *P* < 0.05 was considered significant.

Data from the final question, which was a free-text field with room for general comments or any other additional information, were analysed by one of the researchers (LE) in several steps. First, the whole text was read through for the purpose of becoming familiarised with the data. Second, inductive coding was applied. After the inductive coding was completed, it became apparent that the codes that emerged were very similar to the emerging themes from the thematic analysis of the FGDs. In a third step, deductive coding using the emerging themes from the analysis of the FGD was applied to the data.

## Results

### Focus group discussions

In total, six FGDs were conducted, comprising a total of 25 hunters (minimum three, maximum five participants per FGD) from five different hunting districts (located in the counties of Jönköping, Skåne, Södermanland, Uppsala and Västmanland) (Fig. 1). A few of the participants hunted in other districts outside the study area (namely in the counties of Dalarna, Hälsingland, Jämtland, Västerbotten and Västernorrland). Of the 25 hunters, three were women and 22 were men. This skewed gender balance roughly represents the gender composition of Sweden’s licensed hunters (8.1% female in 2022) [19]. The median age was 47 years (minimum 20, maximum 72 years). The majority of the participants reported that they hunted several times a week.

In the thematic analysis, 46 primary codes emerged (Table 1). Some of these codes were related to each other and could be grouped into eight emerging themes. From the emerging themes, three general topics could be derived. The emerging and general topics are developed and described in the subsequent section.

**Table 1:** Primary codes, emerging themes and general topics from the six focus group discussions with hunters conducted in 2020

**General topics** • Hunters are willing to engage in ASF prevention and control • Feasibility is crucial for the implementation of reporting, sampling and control measures • More information and the greater involvement of the authorities are required in ASF prevention and control	
**Primary code**	**Emerging theme**
Concern about ASF introduction (general) Concern about ASF introduction with imported feeds Concern about ASF introduction with tourists and lorries Hunters are not the major risk of introduction Increased border control for pig/pork/wild boar products Will change practices if ASF arrives	ASF introduction
Carcass detection Carcass finder’s fee – positive and negative perceptions Concerns about ASF spread with carcass handling Finding wild boar carcasses is difficult Hunters are already spending the maximum possible time in forests	Carcass handling
Control measures – generally positive perceptions Control measures need to be easy Culling is not hunting Fencing – difficult and negative perceptions Financial compensation per shot wild boar – positive perceptions Forest access restriction – positive and negative perceptions Incentives to increase hunting Selective hunting – positive and negative perceptions Population density control – positive and negative perceptions	Control measures
Cleaning and disinfection of hunting equipment Promotion of wild boar hunting in Sweden Trophies are important Use of hunting weapon exit passport to enable risk-based information campaigns	Hunting tourism
Hunters’ knowledge gaps concerning ASF Information campaigns to the public Information requirement for hunters Use hunters as ASF ambassadors to inform people about the disease	Knowledge
ASF task force within hunting organisations Contact with authorities Hunters’ local knowledge important Hunters’ work needs to be remunerated (Top-down) coordination and governance	Outbreak management
Relationships with farmers Relationships with other hunters Relationships with landowners	Relationships and cooperation
Better feedback after reports Early detection Form a task force within hunting organisations for ASF reporting Integration with hunting applications Knowledge gaps concerning reporting and sampling Not relying on hunters volunteering for reporting and sampling Online reporting – not aware Positive perceptions of reporting Reporting and sampling need to be easy and simple Use local hunting leaders for reporting	Reporting

#### Emerging themes

##### ASF introduction

The participants expressed a general concern that if the ASF epidemic continued to develop in Eastern/Central Europe, the disease would eventually reach Sweden at some point. This scenario was described as almost inevitable: *“In the long run, I think it’s incredibly hard not to get it. Whether it will be in 3 years, 5 years, 10 years or 20 years I can’t say, but I believe that sooner or later we’ll have it in the country.”* (FGD 3).

The participants expressed concern and worry about the negative impact that the introduction of ASF would have on their own leisure hunting and on the hunting industry. In this regard, several risks of introduction were repeatedly mentioned: hunting tourism including the risk of bringing back equipment, dogs and trophies; workers, truck and ferry traffic from ASF-infected countries in Europe and the associated danger of people bringing and discarding infected pork/wild boar products within reach of Swedish wild boar; and the import of animal feed from affected countries.

##### Carcass detection

Participants said that despite spending a lot of time in the forest (hunting, preparing for hunts, walking dogs, picking berries and mushrooms), they rarely see wild boar carcasses. They mentioned that wild boar carcasses are hard to find as the animals tend to hide in inaccessible terrain. Participants explained that it was literally impossible to spend more time in the forests without compromising work or family duties, especially if there is no compensation for it: *“…every weekend there is a hunt. So we’re out examining our own land thoroughly, basically all the time at weekends”* (FGD1). A more immediate or closer threat of ASF or a specific mission to search an exact area might be an incentive for active carcass searches. The issue of compensation for detected/reported carcasses was discussed and was not really considered to be an incentive (as the participants already spend the maximum possible time in the forest), but it would be seen as a positive gesture, at least compensating for direct costs such as fuel. Several participants said that they were not sure if handling carcasses constituted a risk of spreading ASF, and therefore were unwilling to do so. It was suggested that information about what to do with found carcasses should be included in information materials handed out in connection with larger hunts.

##### Control measures

The participants expressed a generally positive attitude towards contributing to ASF control. This was based on a desire to reduce the negative impact of ASF on the wild boar population, which was regarded as a valuable resource, on hunting as a hobby and a lifestyle, and on other stakeholders involved in forests and farming: “*As wildlife management is more or less our main task, it’s good if diseases are not being spread among animals. It’s not just among pigs, it’s all of them”* (FGD5). In this regard participants seemed to trust that the authorities would know what the most effective control approach would be and expressed their readiness to be involved and assist. However, they emphasised that control efforts cannot rely solely on hunters volunteering to help: *“I think it’s quite considerable, there’s a considerable interest from hunters to help out, so I think that if there were information and training, and even some financial compensation, there wouldn’t be any problems”* (FGD 1) and *“No, but we should be clear that to carry out something like this, it’s not for entertainment, it’s very hard work and takes many hours. We’re counting on people doing this voluntarily. Nobody has the resources to do it on voluntary basis”* (FGD 6).

Some of the specific control measures used in the ASF epidemic in Europe were discussed: culling, fencing, reducing the wild boar population, restricting access to forests, and selective hunting. General restrictions in forest access were considered to be counterproductive as this would mean that wild boar hunting would cease, and population sizes thus increase. They also discussed that if access to forests were restricted in some areas where there is a high density of other wildlife populations that damage crops (e.g. fallow deer), the reduced hunting pressure could potentially result in substantial damage to forests and farming. The abundance of forests and the significance of forests for the general population’s recreation in Sweden were also underlined. Locally applied access restrictions were still considered to have a negative impact on the participants’ daily lives, but would be acceptable for the common good of ASF control and for short time periods: *“But absolutely, if it was swine fever, to not spread that, we would of course do it, but it’s not something that people want to do”* and” *No, then we have to deal with the problem and solve that problem so that we can get out into the woods and the lands”* (both FGD 5).

Fencing was discussed as being difficult to implement. Factors mentioned were the ability of wild boar to penetrate fences, the difficulties of fencing off areas where people live and farm, administrative challenges with multiple landowners and also the length of the fence if the fenced areas needed to include the entire home range of affected populations. Some participants were aware of the positive experience with fencing from the ASF outbreak in the Czech Republic, and seemed to have a more positive attitude towards the use of fencing for controlling ASF.

As for other control measures and reporting (see below), participants were positive about assisting in reducing the wild boar population, but requested clear information both regarding the purpose of the measure and instructions for the procedures for doing so. Many participants had experienced challenges with reducing the wild boar population by hunting (increased hunting pressure leading to dispersal of populations, increasing populations despite intensive hunting, and the time and manpower required). Incentives for increasing wild boar hunting were proposed: reducing the rent for state hunting grounds, reducing the cost for *Trichinella spiralis* and caesium testing, and facilitating and legalising the sale of wild boar meat by hunters. It was discussed that if the goal was to reduce drastically or even eradicate a local wild boar population, methods not currently permitted for regular hunting (e.g. hunting from cars, use of night vision) and increased cooperation between hunting grounds would be needed. In this regard, participants made a clear distinction between culling (as in shooting large proportions of the populations with the help of baits or traps without using the meat, and disregarding the usual hunting ethics) and hunting. The use of poison was discussed, mainly in negative terms both regarding its efficacy and the ethics of it. A specific method to reduce the wild boar population, selective hunting of females, was discussed in both negative and positive terms: *“So in that particular situation it’s not necessarily so wrong to shoot a sow”* and*”No, not if you know that you have the disease in the area”* (FGD 6). The negative perception of this measure dominated the discussion, with many participants referring to hunting ethics: “*If they become ill in an area where there is confirmed swine fever, then you’ll have to remove everything, so it doesn’t matter what it is. But to go out and shoot a sow with piglets today, no, that’s not done”* (FGD 5).

##### Hunting tourism

Awareness of biosecurity measures while hunting in different hunting grounds in Sweden, on hunting trips abroad and among foreign hunters coming to Sweden for commercial hunting was generally considered to be low: *“I think hunters in general maybe don’t really understand, if you haven’t worked with agriculture, that you don’t go from one cow barn to another cow barn in the same clothes, but you wash clothes and shoes because of the disease transmission risk… […] So that knowledge also has to be mentioned and talked about. Because I don’t know anyone who walks from one cow barn to another cow barn without washing properly and changing shoes *etc*., but I’ve never seen a hunter go and wash or change shoes or clothes when going from a hunt in one area to another area. I’ve never experienced that”* (FGD2). It was discussed that this could be associated with a lack of awareness and knowledge, and also that there is a tradition that hunting clothes should not be clean (to conceal the smell of washing powder, for example). In general, it was not considered problematic to clean dogs, clothes, boots or weapons, but clear information and instructions were requested. One group discussed hunting trophies, stating that it was an important part of hunting to be able to bring back trophies. It was suggested that the application process for taking weapons abroad (weapon passport) could be used as a way to distribute risk-based and targeted information to concerned hunters.

##### Knowledge

The participants’ knowledge about ASF varied, from hunters who had attended courses or lectures and were very knowledgeable about the disease and its prevention and control, how to handle suspicions of outbreaks, and the current status of the epidemic in Europe, to those who had little or fragmented prior knowledge. In general, those who were less knowledgeable about ASF were also more unsure how to handle, report and sample carcasses, for example. Specific knowledge gaps were identified and discussed: how ASF is spread, especially how indirect spread can be avoided, and how biosecurity measures during hunting, such as the cleaning of weapons, equipment and dogs, can prevent the spread of disease. As described previously, the participants were positive about participating in reporting, sampling, prevention and control, but demanded more and clearer information about their role and how to act: “*Everything depends on how the responsible organisations and authorities actually reach ordinary hunters and, yes, in fact the general public, explaining how we should handle the issue of African swine fever. That’s where it begins”* (FGD2).

It was repeatedly mentioned that hunters are better informed about ASF than the general public, and that information campaigns should be directed at other actors who use forests, both for recreation and professionally, including in several languages, in different forms and at specific places such as ferry terminals and country borders: *“Because I think we hunters, we’re updated and have the information, but the general public don’t really know about this. And then there’s a lot more information needed. *Via* all channels really, I think”* (FGD2). Mention was made that hunters and hunting organisations have an important role to play communicating within their organisations, but also with the general public. Reaching out to all members within the organisations was mentioned as challenging because not all hunters use e-mail or social media, for example. Hunters have unique knowledge that could be better utilised, but all the actors concerned need to join forces: *“And when we can do that, then we can be well informed, but we need help to be able to be ambassadors for this issue with swine fever, and again, maybe not put all the responsibility on the hunters, but enlist help from the outdoor recreation organisation or other who can also be ambassadors in relation to swine fever”* (FGD 3).

##### Outbreak management

The need to urgently prepare and plan for an outbreak, and the changes that this might require in organisations at several different levels compared with the current set-up, was discussed. In this regard, the establishment of special “African swine fever task forces” within hunting organisations and hunting districts that would be more informed about the disease, take part in preparing action plans and be ready to react to reports of found carcasses was highlighted. It was emphasised that if hunters were asked to participate in hunting (or culling) outside their own hunting grounds in outbreak situations, this would need to be meticulously organised: to make sure that hunting does not contribute to disease spread, and for security in relation to the use of weapons and different hunting traditions in different hunting districts. In this regard, the importance of utilising hunters’ local knowledge of the wild boar population and hunting grounds was emphasised, as was the opposite: that hunters mobilised to participate in eradication operations in other hunting grounds, for example, would not have this local knowledge. The participants again repeated that ASF outbreak management cannot rely on hunters volunteering for intensive and time-consuming tasks such as eradicating wild boar populations from an infected area. There were calls for firm instructions and top-down organisation from the authorities: *“There has to be some authority that deals with it and maybe does not force us out, but engages our help to prevent the spread of the disease where it emerges. Because it has to be local, the disease has to come somewhere first. There has to be some authority, or SVA, well someone who drives the whole thing”* *(FGD 4)* and *“How are we going to get people? Yes, we’re a hunting team, but not everyone is part of the same hunting team. […] as there is always someone who opposes things and then that’s it. So someone has to come here and put their foot down so that it will be possible to carry it out on the day” (FGD 2).* These two citations reflect a generally expressed perception of trust towards the authorities, but one group (FGD 3) held the opposite view, highlighting a distrust, especially among older hunters.

##### Relationships and cooperation

It was acknowledged that different stakeholders have different interests and priorities, i.e. some landowners and farmers struggle with the presence of wild boar and are eager to reduce their density, while hunters generally see them as a valuable resource that they would like to keep and develop. Owing to this, better cooperation and coordination were called for, for example with the selection of crops to optimise hunting and permission to shoot wild boar even if they cross over to another hunting ground during a hunt. Many of the participating hunters were also well rooted in their respective communities, and expressed concern about the negative impact on the farming and forest sectors: *“It overturns everyday life for so many people. For farming and, well, it’s all businesses in the area that will be affected. Something that I think we all have in common, and all organisations no matter what, it’s that we fight for a prosperous rural environment, and it would be absolutely devastating if we had a hot-spot area in Jönköpings län. […] It’s almost like Covid, but times 200 for the wild boar*” (FGD 2). It was further discussed that if an ASF outbreak were to occur, hunters from different hunting grounds would have to cooperate more closely than before, and clear governance from the authorities would be needed to facilitate cooperation and optimise effective hunting in that scenario.

##### Reporting

A generally very strong interest was expressed among hunters about participating in reporting and establishing the cause of death in wild animals found dead: *“I think that generally among hunters there’s a keen interest if you find a dead animal in sending it to SVA for investigation, because we all want healthy wildlife and to map if there is any kind of disease on our land or in our hunting area. So I think that there is already a strong willingness to help if we were to find a dead animal and send it in”* (FGD1). One group presented a conflicting opinion: that the most immediate action would be to shoot any unhealthy-looking animals and get rid of the carcasses by burying or burning them without any extra tasks attached to this, such as reporting, sampling or carrying a carcass to an accessible place: *“If you see a sick animal or an animal acting strangely, then you shoot it and burn it or bury it. That’s how it works”* (FGD 3). Some participants were aware of what to do if they found a dead wild boar and were frequent users of the current reporting system in Sweden (“Rapportera vilt”). Others were unsure about what to do, did not know how to make a report, or what would happen or be requested of them as submitters of a report following a report being made. In this regard, several participants stated that in such situations they would call a local hunting leader who they were sure would know what to do. It was repeatedly expressed that reporting (as well as the ensuing procedures such as sampling, storage and sending in samples) must be as easy as possible if hunters are to participate: *“But what I’d like to see is that it’s easy and smooth and it should go fast because then you’ll do it. If it takes time and is bothersome then you don’t want to spend energy on it”* (FGD 2). It was suggested that sampling material should be stored in central places in the regions for easy access, and reporting of carcasses incorporated into existing mobile applications used during hunting. When suggesting how reporting could be made easier, some participants described functions that are actually included in the current reporting system: *“It would be easier if there were an app where you can report quickly, that takes your coordinates where you are”* (FGD3) and *“As long as it’s easy to send in animals, if there’s good management around it, that you know exactly how to do it, the transport is paid for, then I don’t think there are any doubts that people will send in what they find”* (FGD4). It was mentioned as important for hunters’ willingness to participate in reporting that the objective of the reporting be explained, and that the person making the report received feedback of the results from all submitted reports and samples. Building up a local/regional organisation for reporting and sampling based in the county administrative boards or in hunting grounds (similar to other hunting and wildlife administrations) was also suggested. It was suggested that there could be a dedicated person (in the hunting district or the county administrative board) with expertise and equipment for reporting and sampling, and for the county administrative board to reduce the voluntary work expected of hunters and hunting districts, with this work being undertaken by paid staff instead.

##### General topics

The emerging themes could be synthesised into three general, overarching topics: hunters are willing to engage in ASF prevention and control, feasibility is crucial for the implementation of reporting, sampling and control measures, and more information and the greater involvement of the authorities are required in ASF prevention and control. Throughout all the emerging themes, it was evident that the participants generally had positive views towards the authorities involved in ASF, and were *willing to engage in ASF prevention and control*. Participants considered it their duty to protect and preserve the wild boar population and to be involved for the common good, although of course with some variability in attitudes and their ability to commit. It was concurrently noticed that participants in most cases already invested a great deal, or virtually all, of their free time in hunting and were negative about putting more (formal or informal) responsibility for ASF prevention and control onto hunters, especially without compensation. This issue was associated with a general theme highlighting the importance of the *feasibility of the implementation of reporting, sampling and control measures*, especially with hunters participating as volunteers. Feasibility in this regard includes all measures being easy and quick to perform and information about what to do and any materials required being easily accessible for all hunters at all times. The need for more information and communication around ASF created a separate general theme calling *for more information and greater cooperation in ASF prevention and control*. In this regard, voices promoting firmness and strictness in the authorities’ contingency planning and outbreak management were balanced by a simultaneous call to bring ASF prevention and control closer to the hunters (using hunters as ambassadors, making better use of hunters’ local knowledge, creating ASF taskforces in the local hunting organisations) and for enforcing feedback loops, for example in reporting. This last point would require the authorities not only to issue instructions, but also to involve hunters in the planning of surveillance, prevention and control, share the science and knowledge behind the suggested measures, and use local knowledge to adapt measures to each local setting. The need for more information on ASF featured in all emerging themes, including both practical and technical information on reporting and sampling procedures for example, knowledge about ASF including its spread, prevention and control, and the general purpose of reporting, sampling or control measure.

#### Online questionnaire

In total, 3244 responses were received for the second version of the questionnaire. As questions in the questionnaire could be skipped, not all respondents replied to all questions. Results from questions that turned out to give ambiguous answers, indicating that they were easily misunderstood, or that were answered by too few respondents to draw any conclusions were not included in the analysis (the latter concerned only one follow up-questions which recieved less than 15 responses).

##### Hunting habits

Questions to describe hunting habits included the counties in which the respondents hunted, and if and how often they engage in the hunting of wild boar in Sweden (Table 2).

**Table 2:** Hunting practices, as stated by Swedish hunters responding to an online questionnaire. *No.* number, *WB* wild boar

**No. of counties hunted in**	**1**	**2–3**	**4–8**
Total answers: *n* = 3233	2517 (77.9%)	630 (19.5%)	86 (2.7%)
**WB hunting**	**Yes**	**No**	
Total answers: *n* = 3210	2747 (85.6%)	463 (14.4%)	
**Extent of WB hunting/year**	**Single days**	**7–14 days**	** > 14 days**
Total answers: *n* = 2752	655 (23.8%)	871 (31.6%)	1226 (44.5%)

Most respondents selected just a single county, but hunting in up to eight counties was reported. All 21 counties in Sweden were mentioned, including the more northerly ones where wild boar are not present, indicating that respondents also participated in the hunting of other species.

##### Baiting

More than a third of the questions were related to the concept of baiting. Some of these answers did not concern baiting intended for wild boar, but if the activity took place in an area where wild boar are present it is still relevant for the purposes of this study. Of the respondents (*n* = 3191), the majority had participated in baiting (*n* = 2222, 69.9%). The ensuing questions regarded the extent to which baiting was implemented in the course of a year, how many people were engaged in maintaining the baiting station, and how much feed was used (Table 3). As the question did not specify this as relating to one specific baiting station or location, the answers may reflect the amount used at more than one baiting station.

**Table 3:** Wildlife baiting practices, as stated by Swedish hunters responding to an online questionnaire. *No.* number, *kg* kilograms

**Baiting deployed**	**On a single occasion**	**During one or a few weeks**	**During one or a few months**	**Continuously**	**Do not know**
Total: *n* = 2203^a^	161 (7.3%)	219 (9.9%)	596 (27.1%)	1173 (53.2%)	54 (2.5%)
**No. of persons maintaining it**	**1**	**2–3**	**4–6**	** > 6**	**Do not know**
Total: *n* = 2194^a^	361 (16.5%)	1109 (50.5%)	387 (17.6%)	298 (13.6%)	39 (1.8%)
**Average kg feed used/year**	** < 100 kg**	**100–300 kg**	**300–500 kg**	** > 500–1000 kg**	**Do not know**
Total: *n* = 2177^a^	459 (21.1%)	643 (29.5%)	365 (16.8%)	492 (22.6%)	218 (10.0%)

^a^Respondents who stated participation at baiting and answered the follow-up questions

A multiple-choice question asking for the leading cause or causes influencing the choice of bait feed was answered by 2166 respondents. More than one cause could be selected. The results are displayed in Table 4.

**Table 4:** Reasons for choice of feed for baiting, as stated by Swedish hunters responding to an online questionnaire. *No.* number

Factors influencing the choice of bait feed	No. of answers	% of respondents
Attractiveness for the animals	1248	57.6%
Availability	1148	53.0%
Simplicity of storage and handling	1015	46.9%
Cost	646	29.8%
Feed safety/biosecurity	169	7.8%
Tradition	154	7.1%
Other	108	5.0%
Total number of respondents (*n* = 2166)	5388	

The order of the top four choices remained unchanged when the responses were stratified based on the amount of feed used. For the group stating that they used the least amount of feed, the fourth factor ‘cost’ was followed by ‘tradition’ and then ‘feed security/biosecurity’, whereas the respondents stating the use of larger volumes of feed had a reversed order in the number of selections of those last two choices. The choice of ‘other’ came with a possibility for further specification. While some free-text specifications were repeats of the choices previously made regarding attractiveness, availability and simplicity of use, other common comments were *“selecting healthy and natural feed for the wild animals”*, *“using products to which the animals have access in the wild”* or *“using products that would otherwise have gone to waste*” (fallen apples, by-products from grain harvest), as well as selecting locally produced cereals and peas. Other comments related to the ease of access or to requirements from the landowner with regard to what feed can be used.

A free-text question asking for the main contents of the feed used for baiting was answered by 2157 respondents. The most commonly mentioned ingredient was maize (*n* = 1501, 69.6%), followed by peas (*n* = 614, 28.5%), cereals and pelleted feed (*n* = 520, 24.1%), root crops and tubers (*n* = 147, 6.8%), fruits and vegetables (*n* = 118, 5.5%) and bread (*n* = 55, 2.5%). Thirteen respondents (less than 1%) mentioned using by-products from slaughter. Seven of them specified that the by-products were from hunting, and four mentioned that it was used with the intention of baiting fox. Two respondents mentioned using fish. Maize, peas and cereals where often used in combination. One respondent who stated that they used slaughter by-products from hunting also mentioned using meat from ‘private consumption’, but did not specify if the meat was from their own harvest of game or included other meat products. A similar multiple-choice question on ingredients placed as bait followed. It showed comparable results, and these are included as Additional file 3.

The ensuing question regarded whether any of the baiting feed used originated from outside Sweden. This was answered by 2219 respondents with’yes’ (*n* = 88, 4.0%),’no’ (*n* = 1779, 80.2%) and’do not know’ (*n* = 352, 15.9%). A follow-up free-text question on what type of feed and what country it originated from was answered by 59 of the respondents who previously stated they had used such feed. The most commonly mentioned imported product was ‘maize’ (*n* = 26), followed by ‘fruits and vegetables’ (*n* = 6), while one mentioned ‘cereals or peas’ and one ‘Norwegian salmon’. The countries mentioned were Poland (*n* = 25), Denmark (*n* = 10), Europe or EU (*n* = 3), USA (*n* = 2) and the Baltic countries (*n* = 2). Two respondents cited maize from Hungary and Ukraine, respectively.

One question addressed the use of animal products or food prepared for human consumption for baiting. This question was answered by 2184 respondents, with the absolute majority (*n* = 1924, 88.1%) stating that they had not used such products. Of the 260 respondents who answered that they had used such products, 240 (92.3%) estimated that they constituted less than 25%, while 20 (7.7%) estimated that they constituted more than 25%.

##### Hunting travel

The first question regarding hunting travel asked if the respondent had ever hunted for wild boar outside of the Nordic countries. This was answered by 3074 respondents, with 418 (13.6%) replying that they had done so. These respondents were also among those who hunted in more than one county (Table 2).

A follow-up question on when and where they had been travelling resulted in 413 free-text replies. Of these, 321 respondents left a year or a comment that allowed their travel to be dated as before 2014 (*n* = 123, 38.3%) or in 2014 or later (*n* = 198, 61.7%). In the case of a respondent travelling both before 2014 and after, the latest travel date was used in the analysis. For countries or regions, 411 respondents made 529 country mentions. The countries or regions mentioned by 20 or more respondents were Germany (*n* = 147), Poland (*n* = 140), the three Baltic countries of Estonia, Latvia and Lithuania (*n* = 47), Africa (*n* = 38), Hungary (*n* = 38) and the Czech Republic (*n* = 20). There were six mentions of Norway, Denmark or Finland, all in conjunction with other countries outside of the Nordic countries.

A single-choice question regarding if wild boar products were brought back to Sweden was answered by 420 respondents with the options ‘yes, trophy parts only’ (*n* = 111, 25.9%), ‘yes, products intended for human consumption’ (*n* = 12, 2.9%), ‘no’ (*n* = 291, 69.3%), ‘do not know’ (*n* = 6, 1.4%). Of the 111 respondents who stated that they brought back trophy parts, 110 answered the follow-up question of whether the trophy parts were processed in any way before they were brought back to Sweden: 103 reported ‘yes’ (93.6%) and seven ‘no’ (6.4%). There was a significant association between respondents travelling for hunting before or after 2014 and bringing back trophies or products for human consumption (*p* = 0.013). Of those who travelled before 2014 and who replied to the question of whether they brought back anything (*n* = 120), 39.2% said they brought back products, while of those travelling in 2014 or later (*n* = 194), 25.3% brought back products.

In all, 418 participants responded to a question of whether they had received any information regarding infectious animal diseases, relevant to hunting travel. Table 5 illustrates the responses by category of organiser and includes the 416 respondents who answered both questions. Focusing on those who had travelled in 2014 or later, 85/168 had received biosecurity information.

**Table 5:** Data on hunting travel, as provided by Swedish hunters in response to an online questionnaire

Organiser	Yes, received bio-security information	No, did not receive bio-security information	Unsure if such information was given	Total
Individual person	54	113	9	176
Professional hunting travel organiser	68	101	28	197
Other	12	23	8	43
Total	134	237	45	416

On the question about whether and how clothes and equipment used abroad were cleaned, 68 respondents answered ‘no’ (16.3%), 231 answered ‘yes, basic cleaning, rinsing of boots and visibly contaminated clothing’ (55.5%) and 117 answered ‘yes, thorough cleaning/disinfection, e.g. clothes washed at 60 °C’ (28.1%). Focusing on respondents travelling in 2014 or later, a comparison of whether any cleaning of equipment had been done with biosecurity information provided showed that the odds ratio of cleaning equipment after receiving biosecurity information was 6.22 (CI 1.95- 28.81, *p* = 0.001) compared with those not receiving such information. One question concerned whether the respondent or anyone else participating in the same hunting trip brought a hunting dog from Sweden. In total, 413 replied to this question, with 23 (5.6%) selecting ‘yes’, and 390 (94.4%) ‘no’.

In the next question the respondents were asked whether they had ever invited hunters from abroad to hunt in Sweden, and if so from where and whether biosecurity was discussed or not. This question was answered by 3058 respondents with the alternatives ‘yes’ (*n* = 269, 8.8%), ‘yes, but not to an area where wild boar was present’ (*n* = 93, 3.0%) and ‘no’ (*n* = 2696, 88.2%). Of the respondents who had invited hunters, 271 provided further information regarding country/countries and 33 of these stated that they had provided information about or discussed biosecurity. The countries mentioned by more than ten respondents who had invited hunters were Denmark (*n* = 100), Germany (*n* = 97), Norway (*n* = 36), Finland (*n* = 22) and the USA (*n* = 11).

In the questionnaire’s final comments field, some respondents reflected on answers previously given, while others offered more elaborate answers. In the deductive coding, four out of the eight emerging themes from the FGD analysis were present: “ASF introduction”, “control measures”, “knowledge” and “relationships and cooperation”. Many responses revealed a fear of ASF being introduced, with comments that called for stricter regulations on baiting volumes, and the type and origin of baiting feed used. At the same time, frequent mention was made of hunting at baiting stations being an effective form of hunting, although the amounts used may need to be regulated in order to avoid increasing wild boar populations. There were also mentions of the risk of ASF introduction through wild boar access to rubbish at waste collection centres. Many comments also mentioned tourism, truck drivers and foreign forestry workers as a risk of bringing ASF contaminated meat products that may end up within reach of wild boar in the forest, especially close to service areas or ferry ports. The themes “relationships and cooperation” and “knowledge” were present in responses that emphasised the importance of continuous, reliable information disseminated to relevant stakeholders. Furthermore, many comments included concerns about the current hunting rights system as an obstacle to effective population control. It may contribute to local issues with wild boar density as there are conflicting interests where cooperation between landowners and hunters is needed to prevent agricultural damage.

## Discussion

The results reveal that three years before the outbreak of ASF in Sweden, Swedish hunters were concerned about the disease, seeing it as a threat to the wild boar population, their hunting activities and lifestyle, and most importantly to the local communities of which they are part. One of the major risk factors for ASF introduction that was mentioned in both the FGDs and the free-text responses to the questionnaire was food waste reaching wild boar by means of the careless handling of waste by individuals or at waste collection centres. This is a recognised risk for the introduction of ASF to wild boar populations in ASF-free countries [5], and for Sweden this assumption appears to have been correct as it has been reported that the most probable route of introduction for the outbreak in Sweden was via food waste [17]. In the FGDs it was also evident that the participants’ risk attribution was focused on external groups such as foreign truck drivers and the general public who do not hunt, rather than towards local groups (hunting and farming communities) who were seen as better informed and less likely to introduce ASF through careless handling of food waste [20].

Wild boar hunting tourism to infected countries has been mentioned as a risk activity for introducing ASF to ASF-free countries [15]. The focus group participants expressed worry about hunting tourism as a risk of ASF introduction into Sweden, and the survey confirmed that some Swedish hunters hunt abroad as well as in several different Swedish counties. However, most of the respondents did not hunt outside of the Nordic countries. It appeared that those who did hunt abroad also tended to hunt in more counties within Sweden than their non-travelling peers. About half of the hunters who had hunted outside Sweden since 2014 had received some biosecurity information, and most of them stated that they cleaned their equipment before returning. This, in combination with the results indicating that very few products were brought back from these hunting trips, means that hunting tourism probably does not represent an important threat of exposing Swedish wild boar to ASF. The observed effect on the cleaning of equipment following provision of information on biosecurity in conjunction with travel is positive and shows that providing such information can be useful, despite the generally complex and indirect relationship between increased knowledge and changed behaviour [21, 22]. In addition, it appears that very few people take their dogs on hunting trips outside the Nordic countries.

Baiting/supplementary feeding was not mentioned as a primary risk by the focus group participants, and the survey results confirmed that feed used at baiting stations is rarely of animal origin and that slaughter by-products used for the baiting of foxes mainly originated from hunted game. However, feeding maize was very common and some respondents stated that this must be imported as it is not produced in Sweden. Although this assumption is erroneous, previous imports of maize from Poland were mentioned by a few respondents. Most respondents stated that they only use feed of Swedish origin, including maize. While baiting might constitute a low risk for the introduction of ASF [23], there has been speculation that it might have contributed to the introduction of *Salmonella cholerasuis* into the Swedish wild boar population [24]. Import of pig feed ingredients has been mentioned as a risk for introducing ASF in risk assessments for other countries [25, 26]. Moreover, excessive baiting can maintain wild boar population numbers and influence the animals’ spatial behaviour, making them gather around the baiting station, and is therefore considered a risk factor for disease spread among wild boar [27]. In addition, it is not just baiting meant for wild boar that might present a risk, as wild boar may visit baiting sites intended for other animal species and foxes, for example, may move material from baiting stations to places visited by wild boar.

Early detection is crucial to the management of ASF outbreaks in wild boar, and passive surveillance with testing of all detected wild boar carcasses has been deemed the most effective surveillance component in this regard [5, 28]. Hunters spend a lot of their time in wild boar habitats, and are considered essential stakeholders for early detection and increasing the sensitivity of passive surveillance for ASF [8, 29]. The focus group participants called for more information on how to report dead wild boar and why this is important, and also what is required afterwards of the person making the report. Since the completion of the study, given the development of the ASF epidemic in Europe, a great deal of communication has been provided about the importance of reporting findings of wild boar carcasses and about the online reporting system in use in Sweden (“Rapportera vilt”) aimed at the general public and the hunting community. It would appear that these efforts have been worthwhile; the number of reported wild boar carcasses increased from 36 in 2019 to 76 in 2022. Furthermore, the first detected cases in the current ASF outbreak in Sweden were in carcasses found by local hunters and reported using “Rapportera vilt”.

Once an outbreak has been detected, an active search for wild boar carcasses is needed to map the outbreak and remove the carcasses in order to reduce the environmental contamination [30, 31]. The cooperation of local hunters is essential in this activity [9], as has been seen in the outbreak in Sweden. Although the focus group participants had a positive attitude towards participation in ASF surveillance and control, they also expressed a wish for financial compensation for their efforts or at least for fuel costs. This does not appear unreasonable in light of current legislation regulating compensation for actors participating in eradication efforts in disease outbreaks among domestic animals (Swedish law of epizootic diseases (1999:657) and (1999:659)). Despite hunters’ willingness to contribute to disease control, financial compensation has been identified as an important incentive, and further essential momentum may be lost if there is no compensation framework in place at the start of an outbreak [32]. In the outbreak in Sweden, hunters were compensated for their time devoted to carcass search, although with a slight delay in the system for compensation becoming operational. In this study, a main driver of the willingness to contribute to ASF control appeared to be the feeling that an outbreak and its consequences would have serious negative effects on the participants themselves as well as on their respective local communities. The importance of community cohesion and positive peer pressure in disease control has been recognised for other diseases in other contexts, and shown to be effective for improving the implementation of control or biosecurity measures [33–35]. The significance of access to local forests by the public and landowners described by the participants in the study was confirmed in the ASF outbreak in Sweden, where restrictions severely affected the livelihoods of the local community (unpublished data).

The general awareness of biosecurity around hunting appeared low in both study populations, although the respondents to the questionnaire mentioned cleaning routines. Hunters are the stakeholder group expected to have the greatest knowledge of wildlife management, but this does not necessarily imply knowledge of infectious wildlife diseases or hunters having the same awareness of infectious disease risks and the need for disease prevention that is part of farmers’ everyday life. As hunting is an outdoor event, it can be compared to more extensive livestock keeping, while the toughest biosecurity is generally applied to indoor intensive livestock production [36]. As the hunting community in Sweden and the participants in this study were diverse in age, occupation and education level (although less so in gender), the knowledge about ASF and biosecurity also varied among the participants. Some FGD participants were very well informed and had actively searched for information about ASF prior to the study, whereas others had never heard of it. In the FGDs, the participants said that if they were in doubt about what to do if they found a dead wild boar, for example, they would ask for advice from someone they trusted to be knowledgeable, often the local hunting leader or local SJF representative, underlining the importance of the local community and local knowledge in disease control [37]. Furthermore, the local community with its formal and informal networks provides opportunities for the communication and dissemination of information to individual hunters. This was noted in the ASF outbreak in Sweden where the regional SJF representative and the database held at SJF (viltdata.se) were pivotal for baseline data on hunting grounds and for reaching out to local hunters. The diversity among hunters presents a challenge for communication as the preferred communication channels vary (i.e. some parts of the hunting community do not use e-mail or hunting apps, while others are very comfortable with these channels of communication).

The focus group participants called for stricter governance as well as clear instructions and directions. This can be compared with previous findings about hunters requesting increased participation in wild boar management during outbreaks [10–12, 38], and other contemporary research demonstrating that participation and ownership are pillars of sustainable disease prevention and control [8, 13, 33, 39]. Rather than calling for a “top-down approach”, as in not wanting to be engaged or involved stakeholders in ASF prevention and control, this could however be seen as the hunting community wanting clear instructions for technical issues such as sampling techniques and biosecurity, and requesting more engagement from the authorities in an issue that is very important for them and in which, at the time of the study, they saw a lack of presence of public authorities. For example, participants expressed a fear of ending up without support or an appropriate mandate in situations requiring several landowners and hunting groups to cooperate regarding fencing or local eradication of wild boar populations. In this regard, requests were also made for ASF prevention and control to be brought closer to the hunters, i.e. to increase participation, making better use of hunters’ local knowledge concerning wild boar populations, habitats and hunting.

In this study the combination of focus group discussions and a larger online survey allowed for in-depth insights as well as capturing data from a large number of respondents. Nevertheless, a potential selection bias due to participating hunters being those with a keen interest in the issue and comfortable with online group discussions/online questionnaires cannot be disregarded. In addition, the questionnaire was only distributed to SJF members who have an e-mail registered in their membership profile, meaning that the sample population was biased towards members of SJF and who use e-mail. The focus group discussions included only participants from regions with a wild boar presence, while some respondents to the online survey hunted in regions north of the current extent of the Swedish wild boar population. In the questionnaire, several questions offered an opportunity to provide free-text specification if none of the options were suitable for the respondent (given as “other, please specify”). This field was often selected and used to comment on, or repeat, selected options or leave more general comments on the question. This suggests a general willingness among the respondents to supply detailed information. This study was conducted before the first ASF outbreak in Sweden and thus now provides a unique snapshot of a “before-the-crisis situation” that cannot be re-created.

## Conclusions

Hunting tourism and baiting do not appear to constitute major threats for the introduction of ASF to Swedish wild boar populations. The study participants were generally positive towards the authorities involved in ASF management and were willing to engage in ASF prevention and control. The hunting community is a very important resource for ASF control, and their goodwill may not last if it is not nurtured. In this regard, compensation to hunters should be considered not only during outbreaks, but for other surveillance and prevention services as well. Ensuring that information is accessible for all and that reporting and sampling procedures, for example, are simple and feasible seem to be other important issues for maintaining the positive engagement of hunters in ASF surveillance and control.

### Supplementary Information

Additional file 1. Topic guide used in focus group discussions.

Additional file 2. Translated version of the online questionnaire.

Additional file 3. Questionnaire question not presented in main text due to redundancy.

## Data Availability

All data and materials are available by email to the first author upon reasonable request.
