# Supplementary material for: Perceptions and practices of Swedish wild boar hunters in relation to African swine fever before the first outbreak in Sweden
Source: BMC Vet Res. 2024 Jul 17;20:320. doi: 10.1186/s12917-024-04183-9 (PMC11253465; doi:10.1186/s12917-024-04183-9)
Supplement: Supplementary file 1 — Additional file 1. Topic guide used in focus group discussions. [file 12917_2024_4183_MOESM1_ESM.pdf]

Additional file 1. Focus group discussion topic guide

Translated from Swedish to English for the purpose of this article.

Place:
Date:
Number of participants: Women: Men:
Start time: Finish time:
Facilitator:
Note taker:

Instructions: Introduce yourselves, your roles during the discussion and the discussion rules; explain the purpose of the discussion; ask for permission to take notes, photos and record audio and video; and inform about anonymity and voluntariness.

Background information about participants:

1. Name
2. Age
3. Gender
4. Land owner yes/no
5. Hunting experience (years)
6. Main hunting area

Discussion:

Describe your everyday hunting life in relation to wild boar and wild boar hunting?

We talked about African swine fever before, what are your thoughts about that disease?

What advantages do you see in preventing African swine fever from coming to Sweden?

Have you found any dead or sick wild boar, and what do you do if you do?

What advantages or disadvantages do you see in reporting to SVA if you find a dead wild boar?

What would motivate you to report more/submit more?

What would motivate you to go out into the forest more to actively look for wild boar carcasses?

What can you as hunters do to prevent African swine fever from coming to Sweden?

If not addressed, mention the following:

1. do not bring meat products from an infected country

2. do not leave food waste in reach of wild boar

3. if traveling to an affected country, thoroughly clean the equipment before going home

4. avoid handling wild boar feed or visiting a baiting place on return

What do you see as disadvantages with these measures?

What advantages do you see with these measures?

What do you think about the feasibility of these measures?

How effective do you think each measure is?
