# Supplementary material for: Perceptions and practices of Swedish wild boar hunters in relation to African swine fever before the first outbreak in Sweden
Source: BMC Vet Res. 2024 Jul 17;20:320. doi: 10.1186/s12917-024-04183-9 (PMC11253465; doi:10.1186/s12917-024-04183-9)
Supplement: Supplementary file 2 — Additional file 2. Translated version of the online questionnaire. [file 12917_2024_4183_MOESM2_ESM.pdf]

**1**	**In what county/counties are you hunting?**	□ Blekinge, □ Dalarna, □ Gotland, □ Gävleborg, □ Halland, □ Jämtland, □ Jönköping, □ Kalmar, □ Kronoberg, □ Norrbotten, □ Skåne, □ Stockholm, □ Södermanland, □ Uppsala, □ Värmland, □ Västerbotten, □ Västra Götaland, □ Östergötland, □ Västernorrland, □ Västmanland, □ Örebro
**2**	**Are you hunting wild boar in Sweden**	□ Yes, □ No
**3**	**Approximately, how many days per year are you hunting wild boar?**	□ Single days, □ 7–14 days, □ More than 14 days
**4**	**Have you participated in baiting or supplemental feeding of wildlife, in areas where wild boar are present? (even if the baiting/supplemental feeding was not intended for wild boar)**	□ Yes, □ No, □ No, but baiting stations are kept by others on my land.
**5**	**To what extent is the baiting/supportive feeding used?**	□ Single occasion per year, □ During one or a few weeks per year, □ During one or a few months per year, □ Continuously, □ Do not know
**6**	**How many people use or maintain the baiting station/stations?**	□ 1, □ 2–3, □ 4–6, □ More than 6, □ Do not know
**7**	**What amount of baiting- or supplemental feed is used at the station/stations during a year?**	□ Less than 100 kgs, □ 100–300kgs, □ 300–500kgs, □ 500–1000kgs, □ More than 1000kgs, □ Do not know
**8**	**What composes the main part of the feed used for baiting?**	*Free text*
**9**	**From the list below, please select all alternatives that has been used at the baiting station/stations at any occation (regardless of amount). If “Other”, please specify.**	□ Cereals, □ Fruits, root crops or other vegetables, □ Food intended for human consumption (eg. leftovers from households, restaurants, or food industry) □ Meat/meat products or slaughter by products from wild boar or domestic pigs, □ Commercially produced baiting feed, □ Meat/meat products from other wildlife or livestock, □ Do not know, □ Other:_____
**10**	**Does any of the feed used for baiting originate from a country other than Sweden?**	□ Yes, □ No, □ Do not know
**11**	**Which of the used feed originated from outside Sweden, and do you know which country it came from?**	*Free text*
**12**	**If used, what proportion of the feed used for baiting consisted of meat, other animal products or food intended for human consumption?**	□ Not used, □ Very little, □ Less than 25%, □ 25–50%, □ 50–75%, □ More than 75%
**13**	**What are the leading cause/causes behind the choice of feed used for baiting? If ”Other”, please specify.**	□ Cost, □ Availability, □ Attractivity for the animals, □ Feed safety/biosecurity □ Simplicity of storage/handling, □ Tradition, □ Other:________
**14**	**If you at any point used material of animal origin for baiting, was it heat treated or prepared in a similar way before it was used? If so, please specify**	□ Not used, □ No treatment, □ Yes:_______

**15**	**Have you previously been hunting or are you planning to go hunting wild boar outside the Nordic countries?**	☐ Yes, ☐ No, ☐ No, but I plan to, ☐ Do not know
**16**	**Where, outside the Nordic countries have you been hunting? Which year and month?**	*Free text*
**17**	**Where, outside the Nordic countries are you planning to go hunting?**	*Free text*
**18**	**Who arranged the abroad hunting? If "Other", please specify.**	☐ Private individual, ☐ Professional hunting travel organizer, ☐ Other:_______
**19**	**Do you remember the name of the organizer, if so, please write it in the free text field**	☐ No,<br>☐ Yes, ________
**20**	**Was any part of shot wild boar brought back to Sweden?**	☐ Yes, but only trophy parts, ☐ Yes, products intended for human consumption, ☐ No, ☐ Do not know
**21**	**Was the trophy processed (e.g heat treated) in any way before bringing it back to Sweden?**	☐ Yes,<br>☐ No
**22**	**Did the organizer of the travel hunt give participating hunters any information on infectious diseases?**	☐ Yes,<br>☐ No,<br>☐ Not sure
**23**	**Was any gear (clothes, boots etc.) that could have been contaminated with blood or faecal matter from wild boar brought back to Sweden?**	☐ Yes<br>☐ No
**24**	**Was gear used at hunting abroad cleaned at the return to Sweden?**	☐ No, ☐ Yes, basic cleaning, rinsing of boots and visibly contaminated clothing.<br>☐ Yes, thorough cleaning/disinfection, f. ex. clothes washed at 60°C
**25**	**Did you, or anyone else participating in the travel hunt bring a hunting dog from Sweden?**	☐ Yes<br>☐ No
**26**	**Was the dog bathed at the return to Sweden?**	☐ Yes, ☐ No
**27**	**Have you ever invited foreign hunters to hunt in Sweden?**	☐ Yes, ☐ Yes, but not to areas where wild boar was present, ☐ No
**28**	**Which country were the invited hunters from? If relevant, did you consider and address appropriate biosecurity measures (Clean gear/clothing/dogs, no leaving of food in the forest etc.)**	*Free text*
	**Additional comments:**	*Free text*
