# Supplementary material for: Perceptions and practices of Swedish wild boar hunters in relation to African swine fever before the first outbreak in Sweden
Source: BMC Vet Res. 2024 Jul 17;20:320. doi: 10.1186/s12917-024-04183-9 (PMC11253465; doi:10.1186/s12917-024-04183-9)
Supplement: Supplementary file 3 — Additional file 3. Questionnaire question not presented in main text due to redundancy. [file 12917_2024_4183_MOESM3_ESM.pdf]

Additional file 3

Responses to a multiple choice question on what products they had ever used for baiting or supportive feeding, answered by 2186 hunters.

Product used	No. of answers (%)
‘Cereals’	1716 (78.5 %)
‘Fruits, root crops or other vegetables’	896 (41.1 %)
‘Commercially produced baiting feed’	310 (14.2 %)
‘Other’	225 (10.3 %)
‘Food intended for human consumption (e.g. leftovers from households, restaurants or food industry)’	122 (5.6 %)
‘Meat/meat products from other wildlife or livestock’	64 (3.0 %)
‘Meat/meat products or slaughter by-products from wild boar or pigs’	45 (2.1%)
‘Do not know’	52 (2.4 %)
Total number of respondants: n = 2186	3430

Of the 225 respondents selecting ‘Other’ as food that had even been used for bait, 224 left a free text response containing ‘maize’ (n=115, 51.3%), ‘peas’ (n=47, 21.0%), ‘silage’ (n=28, 12.5%), ‘bread’ (n=27, 12.1%), ‘cereals or pelleted feed’ (n=8, 3.6%), ‘fruit and vegetables’ (n=7, 3.1%), ‘root vegetables’ (n=6, 2.7%), ‘fish’ (n=3, 1.3%) and less than one percent mentioned the use of by-products from slaughter or game killed by traffic (n=2). Some respondents mentioned more than one product, hence the total number of mentions exceed the total number of responses.
